# Under-Representation of Racial Groups in Genomics Studies of Gastroenteropancreatic Neuroendocrine Neoplasms

**DOI:** 10.1158/2767-9764.CRC-22-0093

**Published:** 2022-10-12

**Authors:** Brendon R. Herring, Andrew Bonner, Rachael E. Guenter, Selwyn Vickers, Clayton Yates, Goo Lee, Deepti Dhall, Herbert Chen, J. Bart Rose

**Affiliations:** 1 Department of Surgery, University of Alabama at Birmingham School of Medicine, Birmingham, Alabama.; 2 Department of Biology, Tuskegee University, Tuskegee, Alabama.; 3 Department of Pathology, University of Alabama at Birmingham School of Medicine, Birmingham, Alabama.

## Abstract

Not all populations are poised to benefit from advancing genomics in gastroenteropancreatic neuroendocrine neoplasms (GEP-NEN), as genomics have focused on White patients. This study aimed to evaluate racial populations represented in genomic studies of GEP-NENs and to provide evidence of differential genomic findings between racial groups in GEP-NENs. Manuscripts analyzing DNA, RNA, or DNA methylation in GEP-NENs were queried using PUBMED and EMBASE. NIH race/ethnicity term frequency was then determined by Natural Language Processing, followed by manual evaluation of tumor types and subjects by racial group. IHC of institutional tissue micro-arrays and analysis of AACR GENIE data analyzed was performed to determine mutational differences between Black and White pancreatic NEN (pNEN) patients. 313 manuscripts conducted the requisite genomic analyses, 16 of which included subject race data. Race data were included in 13/184 DNA, 4/107 RNA, and 1/54 DNA Methylation analyses. These studies included 89% White subjects (*n* = 2032), 5.8% Asian subjects (*n* = 132), 4.0% “Other” subjects (*n* = 93), and 1.2% Black subjects (*n* = 27). No Native American/Alaska Native, Native Hawaiian/Pacific Islander, or ethnically Hispanic/Latinx subjects were represented. There were significant differences in *MEN1* mutations among Black and White patients in immunohistochemical (13:40) and GENIE data (24:268 patients per group, respectively), with 9 additional genes differentially mutated in the GENIE dataset. Genomic sequencing data for GEP-NENs is almost racially homogenous. Differences in pNEN genomics may exist between racial groups, highlighting a need for diversity in future genomic analyses of GEP-NENs to understand the putative influence of interracial genomic variation on GEP-NEN prevention, diagnosis, and therapy.

**Significance:**

There is little diversity in genomic studies of GEP-NENs, which may exhibit clinically impactful variation in their tumor biology among racial groups. Improved diversity in such studies is imperative for understanding this variation and its potential impacts on disease prevention, diagnosis, therapeutic targeting, and clinical outcomes.

## Introduction

Gastroenteropancreatic neuroendocrine neoplasms (GEP-NEN) are a heterogeneous group of tumors arising from the enteroendocrine secretory cells of the gastrointestinal tract and endocrine pancreas. Pancreatic (pNEN) and small intestinal (siNEN) NENs are the most common types of GEP-NENs, accounting for 3%–5% and 35%–42% of pancreatic and small intestinal malignancies, respectively (1). Some GEP-NENs secrete bioactive substances (functional tumors) that cause severe symptoms such as debilitating diarrhea, life-threatening glucose imbalances, bleeding ulcers, and heart failure. Surgery is the only curative therapy, but many patients with GEP-NENs have metastases at presentation, making curative resection unlikely (2). Most GEP-NENs are diagnosed between the ages of 55 and 69, although colonic NENs peak after age 70 (3). However, across all GEP-NENs, White patients are diagnosed at significantly older ages compared with all other ethnoracial groups. Notably, an analysis of the SEER database found that the incidence of GEP-NEN varies significantly among ethnoracial groups, occurring more frequently in Black patients (5.19 cases per 100,000 individuals) compared with other ethnoracial groups (White 3.05; ethnically Hispanic/Latinx 2.46; others 2.39; ref. 4). Additional analyses have found significant differences in the rate of metastatic disease among racial groups, with Black patients having the highest rate of advanced stage/metastatic disease in pancreatic, gastric, and appendiceal, NENs (36.3%, 20.9%, and 13.5%), followed by White patients (34% 16.7%, 12.1%), Hispanic/Latinx patients (28.2%, 15.1%, 5.65%), and Asian patients (26.7%, 16.7%, 19.8%; ref. 3). Interestingly, White patients presented with significantly more advanced stage disease in small intestine, colon, and rectal NENs (25%, 40%, 11.3%) than ethnically Hispanic/Latinx patients (25.6%, 27.5%, 6.5%), Black (18.1%, 35.3%, 7.2%), and Asian patients (18.8%, 35.3%, 6.5%), raising questions as to the etiology of these differences in GEP-NENs of various primary sites (3).

Regarding clinical outcomes, recent work has uncovered alarming disparities between White and minority GEP-NEN patients- particularly Black patients (3, 5, 6). Black patients with pNENs are more likely to be diagnosed with late-stage disease, undergo curative surgery less frequently, and have a 20% worse overall survival than White patients (6). However, if Black patients have their tumors resected, they have the same overall survival as White patients (6). It is also well described that larger pNEN size directly correlates to increased risk of lymph node metastasis (LNM) and that Black patients often present with larger tumors (5, 7). Alongside these findings, Black patients have been found to have higher rates of LNM in both siNENs and pNENs (8). Notably, Black patients with pNENs have a 360% higher rate of LNM even at small tumor sizes (< 2 cm) compared with White patients (23% vs. 5%; ref. 9). These data, particularly the large disparity in metastasis of pNENs at smaller tumor sizes, suggest that clinically relevant biologic differences in GEP-NENs may exist between these populations.

There is robust precedent demonstrating differentially mutated, expressed, and regulated genes in various cancers among racial groups, representing potentially targetable differences in tumor biology (10–14). DNA methylation is the most stable and best characterized epigenetic modification, although epigenetic modulation of gene expression can occur through histone modification, regulation by noncoding RNA, and a host of other processes. Interestingly, DNA methylation is known to differ among racial populations at CpG loci throughout the genome in healthy tissue (15). Many of these differences are present even at birth, with significant enrichment for these differences at loci associated with cancers that include lung, prostate, and pancreatic among others (16). Relative to other cancers, pNENs and siNENs are mutationally silent, with epigenetic dysregulation as a prevailing hallmark of these neoplasms (occurring in approximately 75%–80% of pNENs and 70%–80% of siNENs; refs. 17–22). Given this high prevalence of epigenetic dysregulation in GEP-NENs and the known baseline differences in DNA methylation between racial groups, differences in the genetic, transcriptomic, and epigenetic ((epi)genetic) profiles of GEP-NENs between these patient populations are highly likely (11, 15). However, there are extensive disparities in the representation of diverse racial groups across all of genomics, extending into the realm of cancer biology (23). This not only diminishes our ability to specifically study (epi)genetic differences between various racial groups in cancer, but it also prevents minority populations from benefiting from advances in precision medicine, genomic screening, and prognostication. Furthermore, equitably assessing (epi)genetic data across more diverse groups may well help the scientific community to further understand the oncogenesis and progression of various cancers. Despite the increasing number of projects aimed at expanding minority representation in (epi)genetic studies and characterizing racially distinct (epi)genetic states in other cancers, there is no work characterizing the state of diversity in GEP-NEN sequencing efforts. Furthermore, there are no studies specifically evaluating the interracial (epi)genomic variation in GEP-NENs that may be present. Accordingly, this study aims to evaluate the representation of racial groups in studies of GEP-NEN (epi)genomics, and to highlight the importance of improved diversity in such studies by providing evidence that indicates the presence of differential (epi)genetic features among racial groups with these cancers.

## Materials and Methods

### Literature Search and Data Selection

**FIGURE 1:**
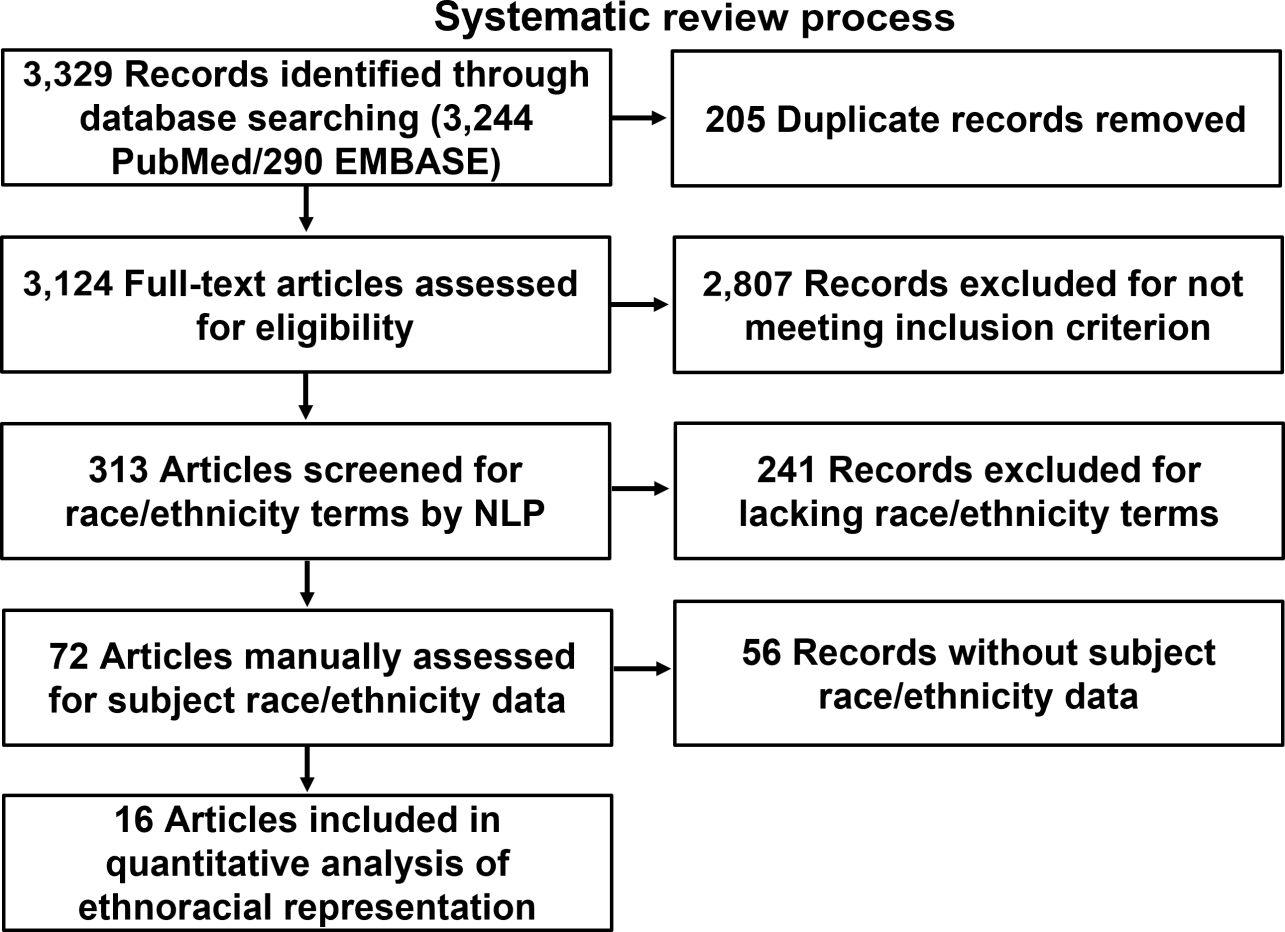
Description of the systematic review process. Studies meeting inclusion criterion for NLP analysis were screened independently by two investigators before screening by NLP and manual review to determine subject number by race.

Literature search was carried out in the PubMed (https://www.ncbi.nlm.nih.gov/pubmed/) and EMBASE (https://www.embase.com/) databases (Fig. 1). Database searches were time-delimited, selecting for publications between the year 2000 until June 2021. Structured search queries included terms pertaining to (epi)genetic analyses of GEP-NENs and are described in Appendix 1 of the Supplementary Data. Articles were included if they met the following selection criteria: published in the English language; conducted in humans; *n* > 1; conducting -omics analysis of DNA, RNA, or epigenetic states (i.e., DNA methylation) in GEP-NENs; conducting array-based or massively parallel next-generation sequencing analysis. Articles analyzing single gene expression, PCR arrays of < 5 genes (not including housekeeping genes), or point mutations at singular base loci (i.e., base 3, *KRAS* G12D/G12C/G12V alone) were excluded. Articles that included only preestablished cell line-based analysis were excluded. Articles such as editorials, letters, commentaries, reviews, clinical practice guidelines, and abstracts from conferences without associated published manuscripts were excluded. Meta-analyses or studies that exclusively studied previously published data, where one would not expect data on subject race to be uniquely presented, were excluded. Cases in which studies included multiple types of neuroendocrine neoplasms (e.g., lung, pituitary, adrenal) were included only if the GEP-NEN component of the study met the inclusion criteria with other NEN types excluded. Furthermore, only the GEP-NEN cases in these studies were included in the analysis of racial/ethnic representation. Studies of mixed neuroendocrine and non-neuroendocrine neoplasms were excluded. Manuscripts focused on pediatric diagnoses were also excluded, as the incidence of all pediatric GEP-NENs is extremely low (under 0.1 per million, excepting appendiceal NENs at 0.5 per million) and these cases may be less likely to conform to the wider distribution of representation across all GEP-NENs (24). Reference lists of all articles meeting criterion were also reviewed for any additional studies meeting criterion. GEP-NENs were classified into 5 groups: pNENs, siNENs, colorectal NENs (including anal NENs), other gastrointestinal NEN (GI-NEN; e.g., appendiceal, gastric, gallbladder, esophageal NENs), and GEP-NENs not otherwise specified (GEP-NEN NOS). Two researchers independently reviewed titles and abstracts manually and selected full manuscripts for inclusion. Disagreements were resolved by discussion and consensus. Manuscripts were reviewed in their entirety, including supplemental materials where present.

### Natural Language Processing

Natural language processing (NLP) using the python packages NLTK (v. 3.6.2) and PDFMiner.six (release 20201018) was used to determine the frequency of the words “Race,” “Ethnicity,” “African American,” “Black,” “Hispanic,” “Latino,” “Latina,” “Latinx,” “Asian,” “Native American,” “American Indian,” “Alaska Native,” “Native Hawaiian,” “Pacific Islander,” “Caucasian,” and “White,” in published original research manuscripts performing sequencing on GEP-NENs gathered by a systematic review of the literature as described above. Specifically, PDF files were read using the extract_text function from PDFMiner.six. Multi-word tokens were then generated using the above race/ethnicity terms using the MWETokenizer function from NLTK. Tokenization and keyword searching was then performed using MWETokenizer.tokenize and text.concordance functions. Natural language processing included supplemental materials where present. Subject numbers by racial group were then determined via manual review following NLP. All manuscripts that were negative for the race/ethnicity terms in our NLP search were likewise manually reviewed to verify that subject race/ethnicity data were not reported.

### IHC Analysis

Protein expression of the *DAXX*, *ATRX*, and *MEN1* genes, which is altered by most mutations, was determined by IHC performed on pNEN tissue microarrays (TMA; refs. 25, 26). Following surgical resection, tumor specimens were fixed, embedded in paraffin and TMAs generated and sectioned by the UAB Pathology Core Research Lab. Slides were rehydrated using xylene and ethanol. Antigen retrieval was accomplished by immersing slides in citrate buffer (pH 6) and placing them in a pressure cooker for 10 minutes. Antibodies to Daxx (Sigma, HPA008736), Atrx (Abcam, ab97508), and Menin (Abcam, ab92443) were diluted at a 1:200, 1:700, and 1:100, respectively, in PBS augmented with 0.3% Tween 20 and 5% goat serum. TMA sections were incubated in primary antibodies overnight at 4°C. Following biotin and peroxidase blocking, sections were incubated with an anti-rabbit biotin–labeled secondary antibody (Pierce goat anti-rabbit IgG, #31820) for 1 hour at room temperature. Slides were then stained with DAB chromogen (Dako Liquid DAB+ substrate) and counterstained with hematoxylin. TMA stains were then evaluated in a blinded manner by a board-certified pathologist specializing in GEP-NENs. All studies of patient-derived tissues were approved by the University of Alabama at Birmingham Institutional Review Board (IRB-300006067).

### Mutational Analysis

Mutational panel data on pNENs were obtained from the AACR's project GENIE database using cBioPortal (27). Patient-level enrichments were determined for protein-altering mutations (nonsense, frameshift, non-start, non-stop, splice-site, and structural variants/fusions including copy-number deletions). Because of the low numbers of non-White subjects included in the AACR GENIE data and the lack of patient samples from other racial groups in our institutional TMAs, only AACR GENIE data from Black or White patients were used in this analysis. In addition, because of potentially confounding differences among sequencing assays used in the GENIE dataset, only sequencing assays that included data for both Black and White patients were included.

### Protein–Protein Interaction Network Analysis

Differentially mutated epigenetic regulatory genes from AACR GENIE (*MEN1*, *KMT2D*, *EP300*, and *SMARCB1*) were used to generate a protein–protein interaction (PPI)-enrichment network using STRING version 11.5 (28). Interaction sources used in PPI network generation were curated databases, experimental determination, text mining, gene coexpression, and gene neighborhoods. Only PPIs with a confidence score > 0.7 based on interaction sources were considered for network generation, and first-shell interactors were limited to 10. Network clusters were determined by k-means clustering. Gene Ontology (GO) functional enrichment of biological processes and molecular functions was also performed using STRING. GO Functional enrichment strength was determined as log_10_ (observed enrichment/expected enrichment), with expected enrichment derived from randomly generated whole-genome background networks of similar size.

### Statistical Analysis

Differences in representation among racial groups relative to their proportions in the U.S. population and cancer genomics as a whole were evaluated by Fisher exact test. Population data for U.S. adults were obtained from the 2020 census (29), while data on the representation of racial groups in cancer genomics was obtained from a recent study of four major cancer genomic studies (TCGA, TARGET, cancer-related GWAS, and the OncoArray Consortium; ref. 23). Mutation frequency and staining were likewise compared between Black and White patients using Fisher exact test. Given the low numbers of samples available from Black patients in the mutational analysis, and the confirmatory nature of the AACR GENIE mutational analysis for our IHC findings, we report significance based on *P* values unadjusted for multiple comparisons. False discovery rate (FDR)-adjusted *P* values are reported alongside *P* values in the mutational analysis as q-values. The mutation comparisons made are reported in Supplementary Data S1. PPI network significance was determined in comparison with expected interactions within randomly generated whole-genome background networks, and *P* values corrected using the FDR (28). Significance of GO functional enrichment was likewise determined. Statistical analyses were performed in R version 4.02 and GraphPad Prism version 8.

### Data Availability Statement

The data generated in this study are available within the article and its Supplementary Data files. Other data used are available within the AACR Project GENIE Database (https://GENIE.cbioportal.org/login.jsp). Further inquiries may be directed to the corresponding author.

## Results

### Manuscript Characteristics

**FIGURE 2:**
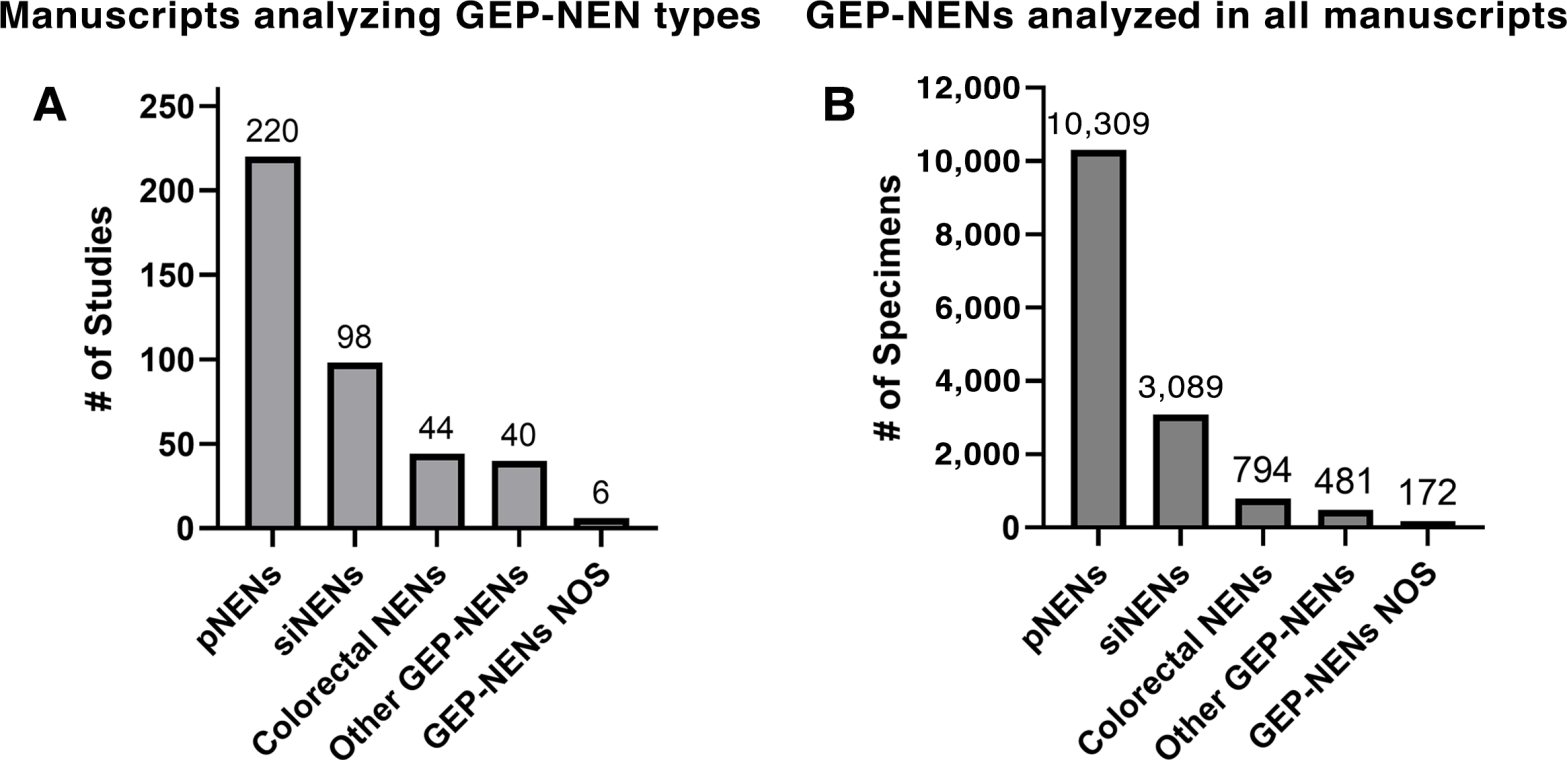
**A,** Number of the 313 total manuscripts meeting criterion that analyzed each GEP-NEN type. **B,** Number of specimens from each GEP-NEN type included in all 313 manuscripts that met inclusion criterion.

Using structured queries as described in the Methods, 3,329 manuscript records were identified (Fig. 1). 205 manuscripts were duplicated across database queries and their duplicates removed from further analysis. 3,124 manuscripts were reviewed for inclusion criterion. Most articles were excluded for not meeting article type criterion (reviews, editorial/opinions, clinical practice guidelines, meta-analyses; *n* = 1438), not performing a requisite (epi)genetic analysis (*n* = 691), or for analyzing only preestablished cell lines (*n* = 212). In total, 313 manuscripts met all inclusion criterion. PNENs were included in most studies (*n* = 220), followed by siNENs (*n* = 98) and colorectal NENs (*n* = 44) (Fig. 2A). The total number of GEP-NENs that underwent (epi)genetic analysis in these studies was 14,845. PNENs were the most abundant GEP-NENs in these 313 studies (*n* = 10,309), followed by siNENs (*n* = 3,089) and colorectal NENs (*n* = 794; Fig 2B). These manuscripts were further subjected to the NLP search strategy, resulting in 72 manuscripts containing our race/ethnicity terms of interest.

### Representation of Racial Groups

**FIGURE 3:**
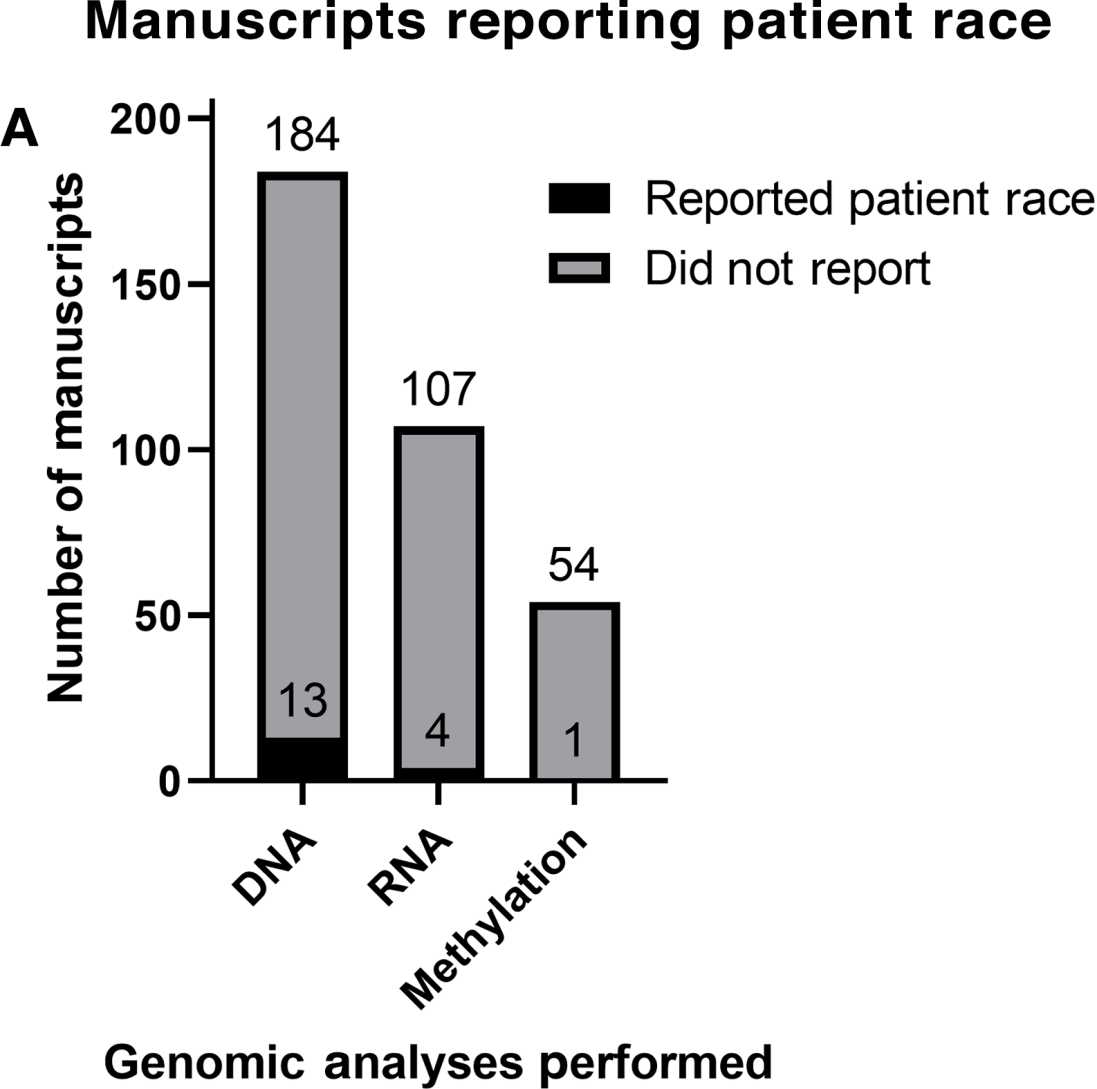
**A,** Number of the 313 total manuscripts that analyzed each type of biomolecule, by the inclusion of patient race information.

**FIGURE 4:**
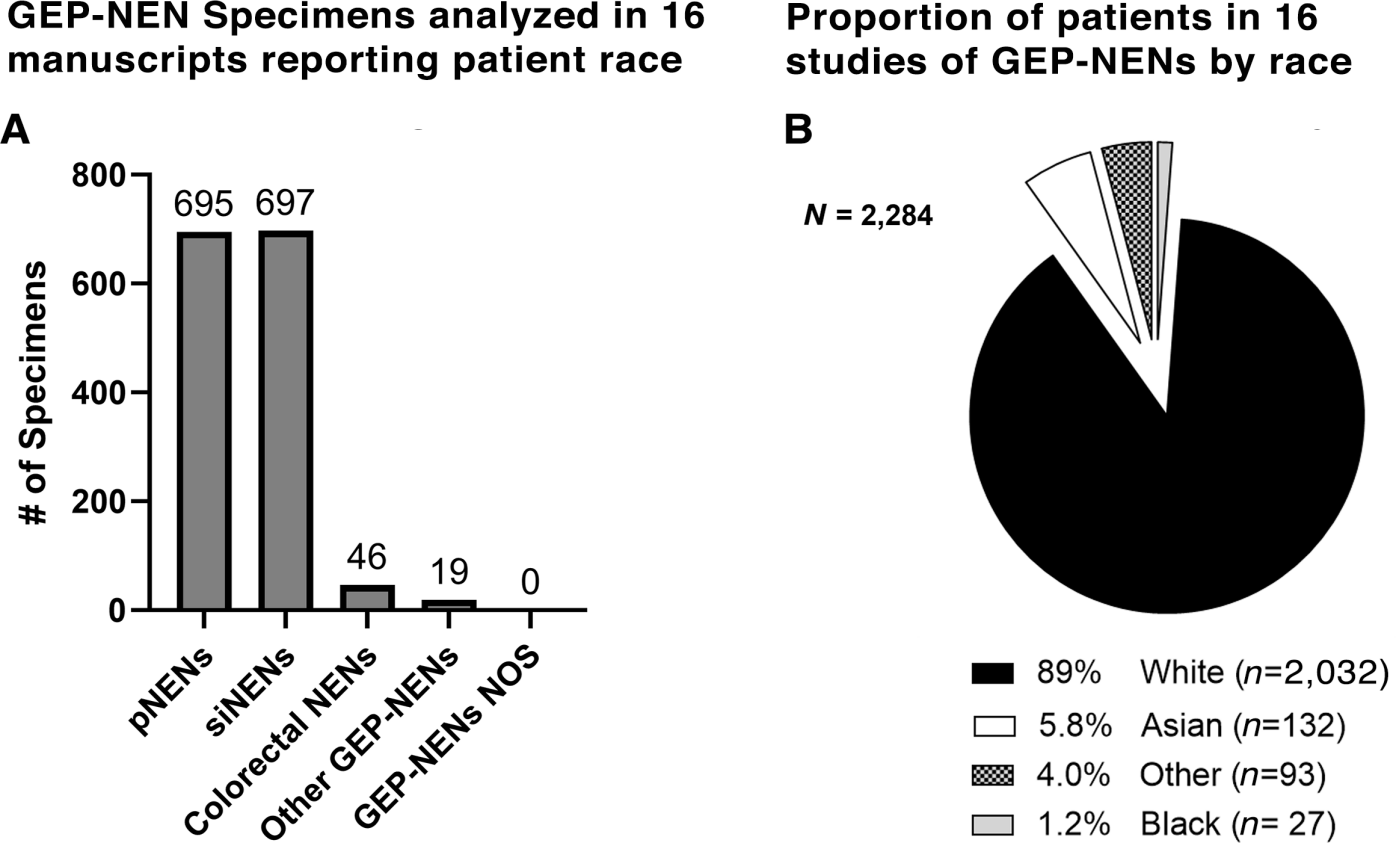
**A,** Number of each type of GEP-NEN specimen analyzed in the 16 studies that reported patient race data. **B,** Number of patients from each ethnoracial group that were included in (epi)genetic studies of GEP-NENs that reported patient race.

In total, 16 of 313 manuscripts included information on the race of the subjects included in their (epi)genetic analyses. Thirteen of 184 studies analyzing DNA included data on the race of their subjects, while 4 of 107 analyzing RNA, and 1 of 54 studies analyzing Methylation included such data (Fig. 3A). The analyses performed in the 16 studies reporting subject race included: SNP and mutational analyses (including genome-wide association studies), targeted NGS/mutational panels, miRNA sequencing, PCR array, methylation-specific PCR, whole-genome sequencing, and gene copy-number analysis (Supplementary Fig. S1). In these studies, siNENs were the most abundant (*n* = 697), followed by pNENs (*n* = 695) and colorectal NENs (*n* = 46; Fig. 4A). These studies included 89% White subjects (*n* = 2032; s = 16, where s = number of studies including White subjects), 5.8% Asian subjects (*n* = 132, s = 8), 4.0% “Other” subjects (*n* = 93, s = 11), and 1.2% Black subjects (*n* = 27, s = 6). No study reported race/ethnicity specific data for Hispanic/Latinx, Native American/Alaska Native, or Native Hawaiian/Pacific Islander subjects (Fig. 4B). The single methylation study that reported patient race included 90% White subjects (*n* = 43) and 10% “Other” subjects (*n* = 5). It should be noted that the discrepancy between total GEP-NENs analyzed in these studies by tumor subtype (*n* = 1,457, Fig. 4A) and the total number of patients by racial group in these studies (*n* = 2,284, Fig. 4B) is primarily the result of studies reporting the race of their entire cohort, while not specifying race information for the subset of their cohort included in (epi)genetic analysis. Hence, although the race representation results in Fig. 4B are as accurate as can be obtained by our methods, they are necessary extrapolations from these whole-cohort demographics and may differ somewhat from the real distribution. Overall, there was a significant difference in the representation of race groups in GEP-NEN genomic studies, relative to their proportion of the United States population in the 2020 Census (*P* < 0.001; Supplementary Fig. S2; ref. 29). White subjects were overrepresented (89.0% vs. 61.6%), Black subjects were underrepresented (1.0% vs. 12%), and subjects from “Other” racial groups were underrepresented (4.0% vs. 9%). Asian subjects appeared to be accurately represented with respect to the 2020 Census data (5.8% vs. 6.1%), but may be underestimated in GEP-NEN genomics due to the use of “Asian” without specific nationality terms in the NLP search strategy. Representation of racial groups in studies of GEP-NENs did not differ significantly from that across cancer genomics as a whole (*P* = 0.27) (23). While the publication dates of studies/databases used in the referenced studies do not differ widely (GEP-NENs 2003 and 2010–2020; aggregate cancer genomics 2007–2016), the aggregate cancer genomics data included pediatric data which may influence this result. Regarding the regional populations studied, most of the manuscripts that reported subject race data studied populations in the United States (6/16, 5/16 exclusively in the Northeast United States), 3/16 studied European populations, 3/16 studied populations in East Asia, 2/16 studied populations in both Europe and the United States, and 2/16 studied worldwide populations.

### IHC Analysis

**FIGURE 5:**
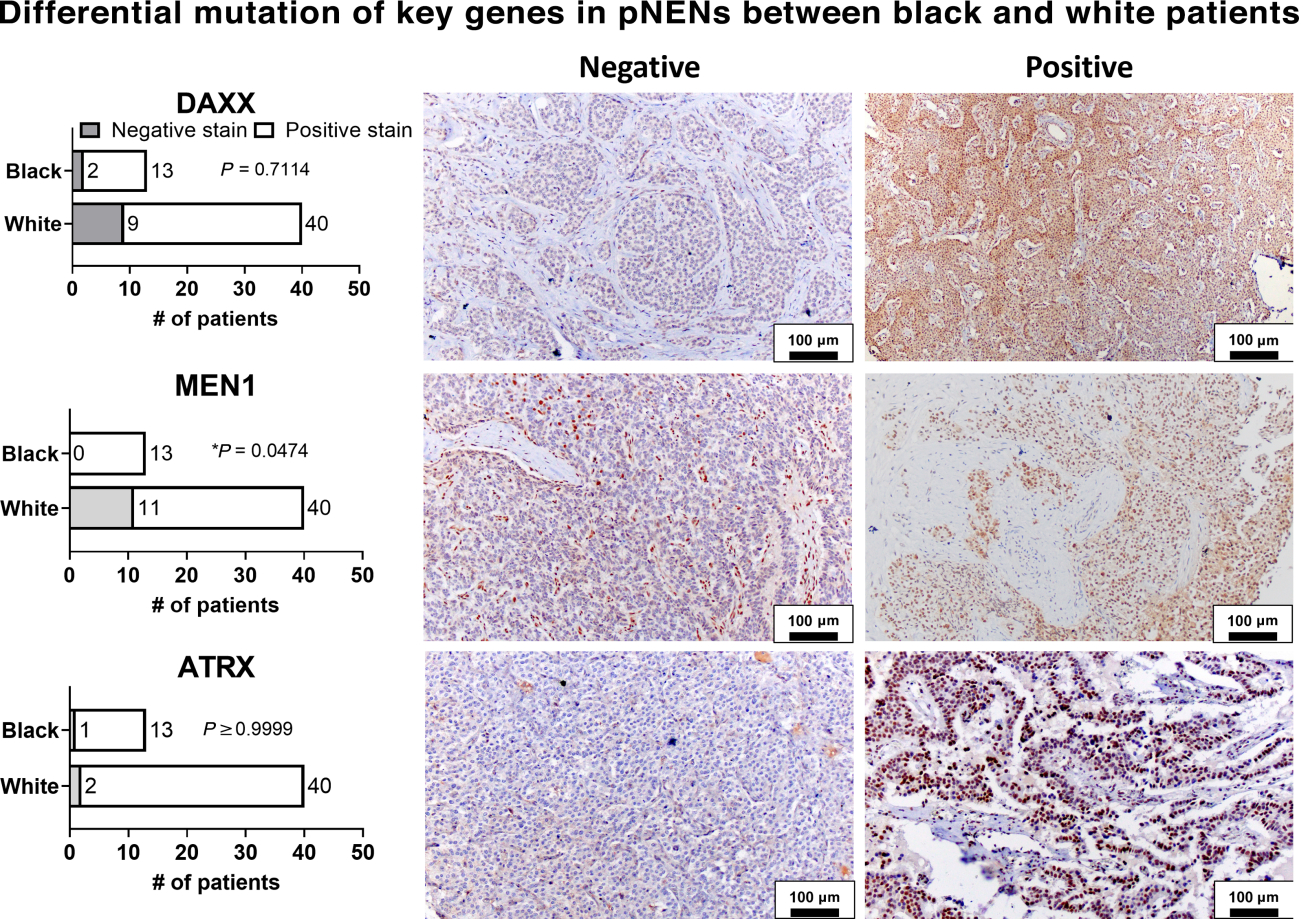
IHC analysis of pNEN TMAs containing tumor specimens from 13 Black and 40 White patients. The frequently mutated genes *DAXX, ATRX,* and *MEN1* were assessed for differential rates of mutation among groups.

Given the low representation of non-White racial groups in GEP-NEN (epi)genomics, we began to question the applicability of previous findings to these groups. As pNENs were the most analyzed tumor in GEP-NEN (epi)genomics, and the genes *MEN1*, *DAXX*, and *ATRX* are the most frequently mutated in pNENs, we investigated their mutational status across racial groups via IHC analyses. Negative staining on IHC indicates a protein-altering mutation, and has been shown to be highly concordant with mutation in these genes (25, 26). Preexisting institutional TMAs containing samples from 40 White and 13 Black patients with primary, well-differentiated grade 1 and 2 pNENs were evaluated (Fig. 5). Nine of 13 (69.2%) Black and 22/40 (55%) White patients were female. Median age at resection (range) were 64 (35–93) and 64.5 (31–82) years for Black and White patients, respectively. Likewise, 6/13 (46.1%) and 24/40 (60%) tumors were grade 1 for Black and White patients. Regarding the IHC analysis, 9/40 (23%) White and 2/13 (15%) Black patients were negative for Daxx expression (*P* = 0.711), 2/40 (5%) White and 1/13 (7%) Black patients were negative for Atrx expression (*P* > 0.999), and 11/40 (28%) White and 0/13 Black patients were negative for Menin expression (*P* = 0.047). Previous studies have found loss of Daxx, Atrx, and Menin expression by IHC in pNENs to occur in 59%, 25%–85%, and 18%–72% of cases, respectively (30–32). The retention of normal Menin staining in specimens from Black patients supports the hypothesis that differential epigenetic modulation may be present in this population (32).

### Mutational Analysis

**FIGURE 6:**
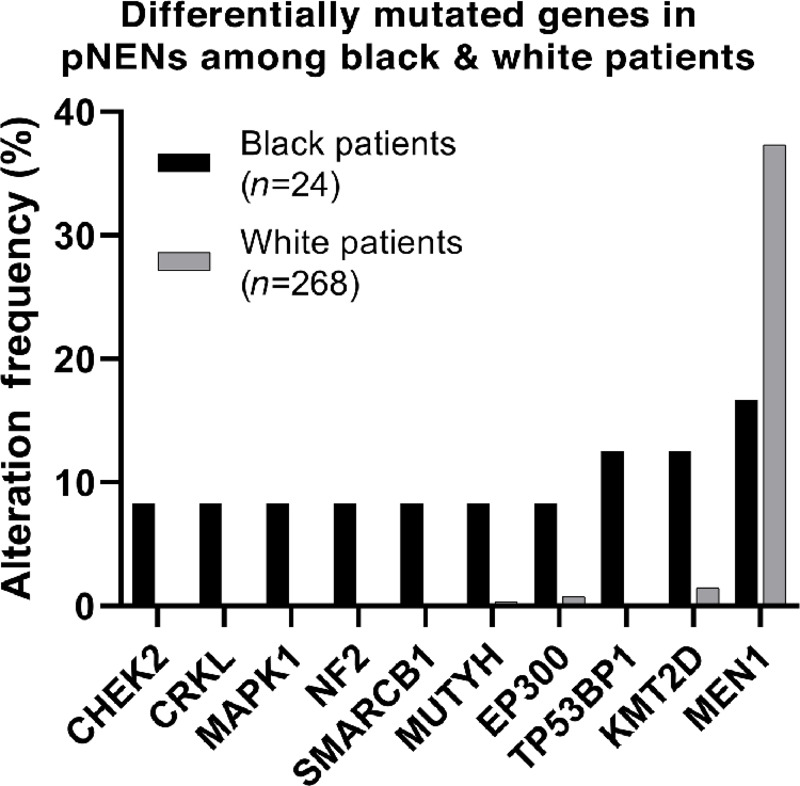
Analysis of mutational panel data from the AACR Project GENIE, comparing the incidence of significant differential protein-altering mutations between Black and White patients with pNENs.

To provide further rationale for the inclusion of diverse subjects in genomic studies of GEP-NENs, we investigated suspected mutational differences between racial populations by querying the AACR GENIE database, which contains publicly accessible mutational data from 28 different institutional sequencing panels conducted on over 400 well-differentiated pNENs from Black and White patients (*n* = 24 and *n* = 399; ref. 27). PNENs were chosen for follow-on studies, as they were the most prevalent neoplasms in the earlier reviewed sequencing analyses. Data from both Black and White patients together were available from nine sequencing assays (Supplementary Fig. S3), consisting of 24 Black and 268 White patients that were included in further analysis. Median age (range) at sequencing were 55 (31–79) and 60 (19–85) years for Black and White patients, respectively. Samples were confirmed metastases in 45.8% of Black and 42% of White patients. Tumor grade was not available. We discovered 10 significant differentially mutated genes in pNENs between Black and White patient groups (Fig. 6; Table 1), including *CHEK2* and *MUTYH* (DNA repair), *NF2* and *TP53BP1* [tumor suppression (TS)], *KMT2D* and *EP300* [histone methyl- and acetyl- transferases (HMT, HAT) respectively], the *CRKL* and *MAPK1* oncogenes, and *SMARCB1* (part of the n/npBAF SWI-SNF chromatin-remodeling complexes). Most notably, we found profound differences in the rate of *MEN1* mutations (TS, HMT), with White patients having a much higher rate of *MEN1* mutation compared with Black patients (37.3% v. 16.7%, *P* = 0.031). Importantly, 4/10 genes found to be differentially mutated have direct roles in epigenetic regulation (*KMT2D, EP300, SMARCB1, MEN1*), supporting previously described differences in epigenetic aberrations between racial groups in cancer (33–37).

**TABLE 1:** Differentially mutated genes in pNENs among Black and White Patients in AACR GENIE dataset

Gene	Black	White	*P* ^a^	Q-val^b^	Relevant function
*TP53BP1*	2 (12.5%)	0 (0.00%)	0.005	0.35	Tumor suppressor
*CRKL*	2 (8.33%)	0 (0.00%)	0.006	0.35	Proto-oncogene
*MAPK1*	2 (8.33%)	0 (0.00%)	0.005	0.35	Proto-oncogene
*CHEK2*	2 (8.33%)	0 (0.00%)	0.005	0.35	DNA repair
*NF2*	2 (8.33%)	0 (0.00%)	0.005	0.35	Tumor suppressor
*MUTYH*	2 (8.33%)	1 (0.37%)	0.019	0.75	DNA repair
*SMARCB1*	2 (8.33%)	0 (0.00%)	0.006	0.35	Chromatin remodeling
*KMT2D*	3 (13.04%)	4 (1.49%)	0.014	0.63	HMT^c^
*MEN1*	4 (17.39%)	140 (40.35%)	0.031	0.95	Tumor suppressor, HMT
*EP300*	2 (8.33%)	2 (0.75%)	0.035	0.95	HAT^d^

^a^Fisher exact test, unadjusted for multiple comparisons.

^b^Q-val = FDR-adjusted *P* value.

^c^Histone methyltransferase.

^d^Histone acetyltransferase.

### PPI Network Analysis

**FIGURE 7:**
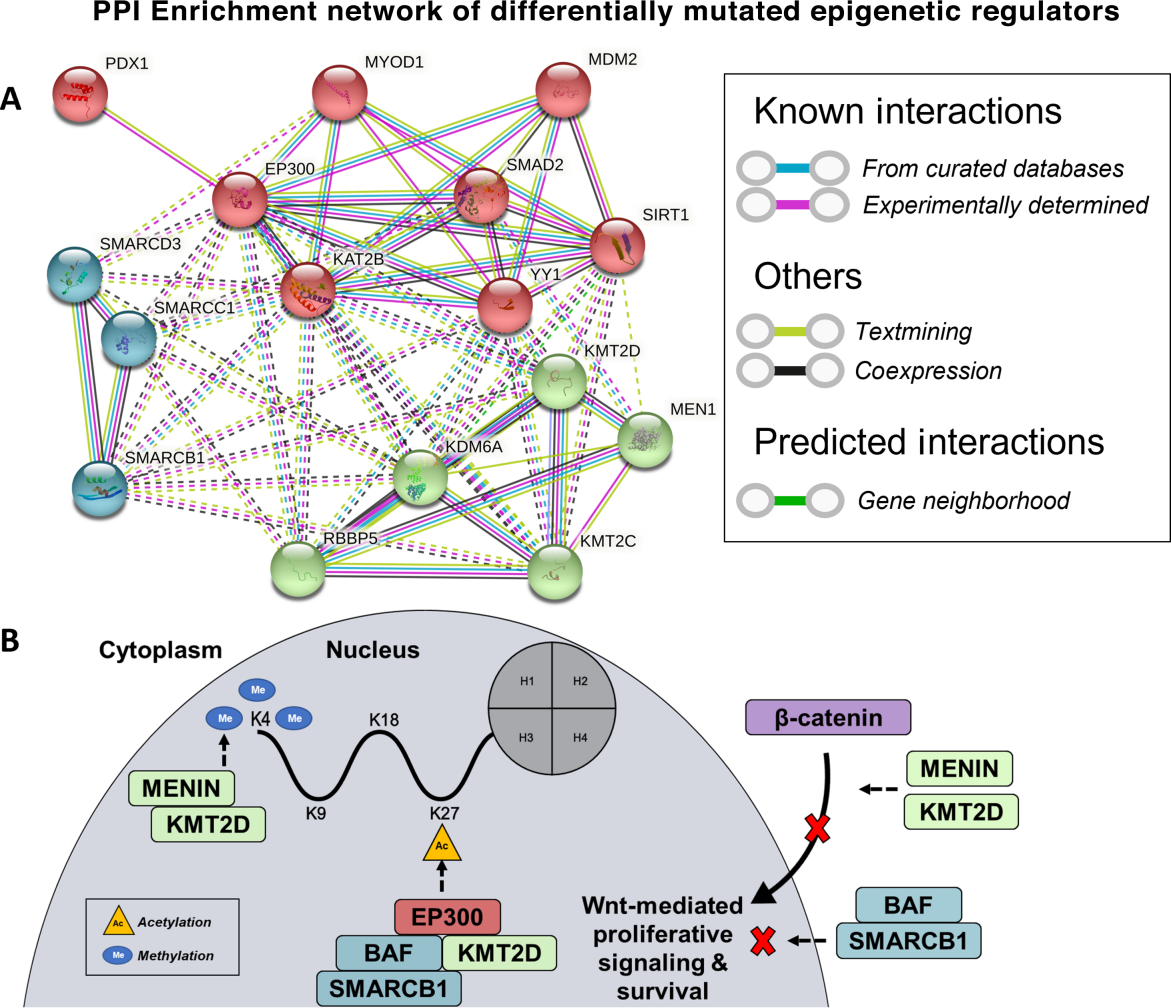
**A,** STRING interaction network of Menin, KMT2D, SMARCB1, and EP300. Between-cluster interactions are denoted by dotted lines, with intracluster interactions denoted by solid lines. **B,** A potential model of functional interdependencies and convergences of Menin, KMT2D, EP300, and SMARCB1 based on network interactions and literature review.

Given known differences in epigenetics between racial groups, a PPI-enrichment network of the differentially mutated epigenetic regulatory genes *MEN1*, *KMT2D*, *EP300*, and *SMARCB1* was generated using STRING (Fig. 7A; ref. 28). The network was highly interconnected and significantly enriched for PPI's (*P* = 2.11e^−15^), with a total of 132 PPI's discovered within the network and a mean of 8.25 interactions per node (Supplementary Data S2). Notably, most PPI's fell within the very high (0.9–1.0) and high (0.7–0.9) confidence score categories (48.5% and 18.2%), with fewer in the medium confidence score category (33.3%; 0.4–0.7). *EP300* had the highest number of interacting partners (*n* = 14), followed by *KAT2B* (*n* = 13) and *KDM6A*/*YY1* (*n* = 11 each). *KMT2D*, *SMARCB1*, and *MEN1* each had 9, 8, and 6 interacting partners, respectively. Gene ontology (GO) enrichment analysis was then performed for biological processes, molecular functions, and cellular components, discovering 196 significantly enriched GO terms in our PPI network (Supplementary Data S3). Notable enriched GO terms included histone H3-k4 methylation, histone acetylation, β-catenin–TCF complex assembly, type b pancreatic cell differentiation, epigenetic regulation of gene expression, chromatin remodeling, MLL3/4 complex, and the nBAF/npBAF complex.

According to these data, we propose an interaction model of the epigenetic regulatory genes found to be differentially mutated between Black and White patients with pNENs in our analysis (Fig. 7B). KMT2D is targeted by the Menin protein (encoded by the *MEN1* gene), forming the MLL3/4 histone methyltransferase complex that regulates H3K4me3 deposition in promoter regions throughout the genome, as well as interacts with the p53 pathway through many of its target loci (38). The histone H3K27 acetyltransferase EP300 requires KMT2D for its binding to a variety of enhancer regions and the facilitation of enhancer-promoter looping, tying mutations in these genes to one another functionally (39). Furthermore, KMT2D has also been shown to associate with the SWI/SNF chromatin remodeling complex, acting as a coactivator for nuclear hormone receptor–driven transcriptional activation (40). This function further involves a core subunit of the SWI-SNF p/npBAF complexes SMARCB1. Of additional note is that SMARCB1 facilitates the effective activation of cell type–specific enhancers by KMT2D and CBP, and CBP closely associates with EP300 for H3K27 acetylation. The BAF complexes/SMARCB1, Menin, and KMT2D all share additional roles in their inhibition of canonical Wnt signaling, thereby acting in a tumor-suppressive manner (41, 42).

## Discussion

We demonstrate herein that few studies on the (epi)genetics of GEP-NENs include data on the race of their subjects. We find that pNENs and siNENs make up the greatest proportion of manuscripts’ topics and tumor specimens analyzed in the literature, as would be expected given their incidence compared with other GEP-NENs. We also find that GEP-NEN (epi)genomics overrepresents White subjects relative to the proportion of the U.S. population that they comprise (Supplementary Fig. S2; refs. 23, 29). Indeed, based on the U.S. population, the expected representation of White subjects is approximately 62%, exceeded herein by approximately 29%. In contrast, Black subjects are represented at approximately 10% of their expected proportion (1.2% vs. 12%). While these findings align with those from other studies characterizing the representation of racial groups in cancer genomics, no Native American/Alaska Native, Native Hawaiian, Pacific Islander, or Hispanic/Latinx subjects are explicitly represented in current studies of GEP-NEN (epi)genomics (23, 43). From these data, we can conclude that there is little representation of racial minorities in (epi)genetic studies of GEP-NENs. Following this, we discovered differences in the mutation of the *MEN1* gene between Black and White patients by IHC. We then conducted a pilot study of differentially mutated genes between these groups using AACR GENIE data wherein we affirmed this finding and discovered 9 other differentially mutated genes. We then conducted PPI-enrichment network analysis on a subset of epigenetic regulators within those genes, finding them to be functionally interconnected, and propose a model of their key interrelated functions. According to the current genetic epidemiology of pNENs, *MEN1* is the most frequently mutated gene, followed by *DAXX* and *ATRX*. The data herein indicate that this may not be the case for different racial groups, warranting more comprehensive analyses of GEP-NEN (epi)genomics among racial groups to identify genomic aberrations that may be enriched in or unique to these populations.

The inclusion of diverse patient populations in (epi)genetic studies of cancer is crucial to understanding and rectifying cancer health disparities, as well as further understanding the biology of various cancers. Therapeutic susceptibility is one important translational aspect of inter-racial (epi)genetic variation that highlights the need for diverse patient representation in sequencing analyses. For example, a phase III trial of the tyrosine kinase inhibitor (TKI) gefitinib for non–small cell lung cancer (NSCLC) found no benefit for any population other than those of Asian descent. EGFR mutations were then discovered to be far more prevalent in Asian patients with NSCLC compared with those of European descent (47% vs. 15%) (44). This led to increased use of TKI's targeting EGFR in this population as first-line therapy, in addition to the discovery that certain TKI's demonstrated increased efficacy in this population (45, 46). There is similar potential for targetable racial differences in sporadic insulinomas (the most common functional pNEN) as well. For example, mutations in the *YY1* chromatin remodeling gene are heavily enriched in Asian patient populations (30% vs. 13%; ref. 47). YY1 is a direct target of mTORC1, inhibitors of which are among the few approved therapies for pNENs, prompting suggestion that this population may uniquely benefit from mTOR inhibitors such as everolimus (48). Furthermore, *MGMT* hypermethylation (present in 17%–50% of pNENs) has been shown to predict response to alkylating agents such as temozolomide, wherein understanding differences in this hypermethylation in Black patients might have immediate clinical implications (49, 50). These points considered, the ability to identify such genomic differences across populations is currently limited by the lack of diverse groups in genomic studies of GEP-NENs, as depicted herein.

The establishment of prognostic genomic features, which allow clinicians to inform treatment selection and conduct prognostication, is an important element of cancer genomic analysis wherein a lack of diverse subjects may exacerbate racial disparities in clinical outcomes. Numerous prognostic genomic features have been characterized as predictors of survival for pNENs, including mutations in *DAXX*/*ATRX* and *MEN1*, the expression levels of somatostatin receptors 2 and 5, elements of the tumor immune microenvironment, and enzymes involved in hormone metabolism (51, 52). However, it is likely that studies characterizing these various features as predictors of disease outcomes reflect the populations included in the (epi)genomic studies reviewed in this study. This is highlighted by our findings that the *MEN1* gene was among those that were differentially mutated between Black and White patients with pNENs. However, while it may be that well-known genes such as *MEN1* for pNENs are not altered in diverse populations, it is possible that “noncanonical” genes representing key nodes of frequently altered pathways are preferentially affected in different racial populations. For example, oncogenic dysfunction of the Menin/MLL4 histone methyltransferase complex in pNENs may occur more frequently via *KMT2D* mutations as opposed to *MEN1* mutations in a given population. This and similar scenarios would entail that such aberrations are identified and included in future studies of association with patient outcomes, as well as clinical screens, to serve a more diverse population's needs adequately and equitably.

Another relevant issue in the diversification of cancer genomics is the exclusive use of self-reported race (SRR) as opposed to the inclusion of genetic ancestry when characterizing racially enriched genomic features. SRR is relatively easy to obtain while also being correlated with genetic ancestry; and its use has persisted in large-scale genomic analyses as a result. Furthermore, SRR acts as a surrogate metric for a highly complex array of behavioral, cultural, environmental, and social variables that are themselves influential in disease (53). However, when SRR data are available they can be inaccurate and incomplete, potentially leading to spurious associations between racial groups and genomic features (54). Although few studies of genomic differences in cancer have incorporated genetic ancestry, broadening knowledge of their influence on tumor biology is driving wider adoption of the practice (55, 56). Numerous tools have been developed that allow for the incorporation of genetic ancestry into genomics analyses, such as Admixture (57) and STRUCTURE (58). These tools use distinct methods to provide maximum-likelihood estimation of individual ancestries from multi-locus single nucleotide variants, or use Bayesian techniques to assign individuals to a predefined *k* number of racial groups based on their genetic features, respectively (54, 59–61). Genetic ancestry may be particularly important to consider for disaggregating racial groups that can become “invisible” in studies of genomics and racial disparities due to relatively low subject numbers, population admixture, discrepancies between SRR and genetic ancestry, or emphasis on studying disparities in certain minority groups over others (62). In addition, the use of genetic ancestry in the conduct of (epi)genomic analyses among racial groups, while not optimal, provides a method for circumventing the remiss practice of aggregating subjects from minority racial groups into an “Other” category without any additional data. Given the complexity of factors associated with SRR, there is ongoing discourse as to if and how genetic ancestry should be incorporated with SRR, rather than considered separately (53, 63, 64). However, it is clear that genetic ancestry should be considered in genomic analyses of diverse populations.

Numerous obstacles have likely led to the current state of diversity in GEP-NEN genomics. Approximately 60% of patients with pNENs have metastasis upon presentation, precluding a curative resection that would generate tissue for biobanking. Alongside this, Black and other non-white patients are less likely to undergo surgery for GEP-NENs, even when they meet societal guidelines for surgical resection and are good surgical candidates (3, 65). Understandably, this results in fewer patient tumor samples from these racial groups being banked for use in sequencing analyses. Alternatively, given that tumor biobanking generally takes place at academic institutions with high surgical volume and that racial minorities are less likely to undergo cancer surgeries at such high-volume centers, these differences likely influence the biobank composition where GEP-NEN (epi)genetic research is conducted (66). A recent analysis of the SEER-Medicare database found that most patients with GEP-NENs receive surgery at medium or high-volume centers (67). However, they also found no significant difference in the racial composition of those treated across hospital volumes. Unfortunately, this study included a relatively small sample size of patients with mixed surgically and medically treated patients (*n* = 899) that was constrained by the availability of completed insurance claim data. As this is the only study analyzing the impacts of treatment center volume on GEP-NEN outcomes to the authors’ knowledge, how the catchment populations of high-volume GEP-NEN research hospitals might be affecting the racial representation in GEP-NEN genomics remains unclear.

Various initiatives and strategies have been aimed at improving the diversity and representativeness of cancer genomics, with focuses on research infrastructure, clinical trial design, community engagement, and researchers themselves. Arguably, the most impactful strategy is the prioritization of inclusive research by institutions, as this manifests as the former through specific goals and increased investment. Increasing the diversity of the cancer genomics workforce is the goal of the AACR's Minorities in Cancer Research Council and the National Cancer Institute's Partnerships to Advance Cancer Health Equity program, which aim to provide training, career development, and research funding for early-stage investigators and trainees from under-represented groups. Improving the diversity of the cancer research workforce may also improve the involvement of diverse groups in clinical trials and genomics studies, as researchers are motivated to address cancer health disparities in their communities and better understand the systemic, logistical, and cultural barriers they face (68). Clinical trials are a key source of (epi)genomic data, as they often involve the collection of patient blood or tumor samples for (epi)genomic analyses alongside therapeutic regimens. Diversity in these clinical trials suffers immensely from the barriers faced by racial minority populations, as they are often confined to large research institutions that may be unreachable, require multiple visits, have restrictive inclusion criterion, have hidden costs of participation, or are distrusted by ethnoracial minority groups. In 2020, the FDA released guidance to facilitate the involvement of underrepresented ethnoracial groups in clinical trials through Project Equity, which included recommendations for decentralizing clinical trial procedures into community facilities, relaxing inclusion criterion, and setting specific goals for enrollment of subjects from ethnoracial minority groups. In tandem with clinical trials, biobanking is a critical aspect of diversity in cancer genomics studies. However, in addition to lacking ethnoracial diversity, data show that biobank donors tend to be both healthier and wealthier than the populations they ostensibly represent (69). Even small improvements in the numbers of subjects from ethnoracial minority groups have been able to improve the detection of genomic variants associated with disease processes, emphasizing the benefits of improving biobank diversity (70). This might be accomplished by using online consent models to facilitate the ease of consenting to biobank participation (71). Such systems also allow institutions to provide information as to how patients’ samples will or are actively being used, which has been demonstrated to be a key factor in decision-making for biobank participation across racial groups (72). Decentralization, or the establishment of multi-institutional regional biobanks that source from community institutions, may be of particular benefit to ethnoracial diversity in GEP-NEN genomics, due to their relatively rare nature (73). While decentralization may also benefit ethnoracial diversity in biobanking similarly to clinical trial enrollment, consistency and standardization of procedures must be carefully considered to ensure specimen and data quality (74).

There are several limitations of the present study to be considered. First is the lack of precise data on the number of patient samples by racial group that underwent (epi)genetic analysis, highlighted by the discrepancy between the total numbers reported in Fig. 4A (*n* = 1,457) and Fig. 4B (*n* = 2,284). Because race representation was reported for whole cohorts rather than for cohort subsets that were included in (epi)genetic analyses, the results in Fig. 4B are an inexact depiction of the unobtainable real distribution. A minor limitation of this study lies in the lack of a specific distribution of subject ages in the comparisons of racial representation in GEP-NEN studies, aggregate cancer genomic studies, and the US population data from the 2020 census. Notably, only data from aggregate cancer genomics studies included pediatric subjects, but specific age distribution data were not readily available in these data and were both variably reported in the GEP-NEN studies that reported patient race data. Another limitation lies in the relatively low number of Black patients compared to White patients in our analysis of pNENs in the AACR GENIE cohort (*n* = 24 and *n* = 268, respectively) and our IHC data (*n* = 13 and *n* = 40, respectively). While this disparity in representation illustrates the points made within and the statistical approach aimed to mitigate differences in sample size, these factors must be considered in the interpretation of our results. Accordingly, it is noted that unadjusted p-values are used herein to determine the number of significantly differentially mutated genes. The small number of subjects in the Black AACR GENIE cohort and the number of genes analyzed (*n* = 323; Supplementary Data S1) rendered all analyses nonsignificant with traditional multiplicity adjustments (Table 1). However, while caution should be exercised in interpreting these data, the lack of MEN1 mutations in the Black AACR GENIE cohort parallels our observations in an independent cohort and strengthens this conclusion. Furthermore, as evidenced by the literature review conducted herein, the AACR GENIE dataset remains the only available repository of data upon which such analyses can currently be conducted, further demonstrating the need for additional studies of underrepresented racial populations in GEP-NEN genomics.

Our understanding of (epi)genetic variation among racial groups has important implications for our understanding of GEP-NENs, and may highlight genomic differences among racial groups influencing oncogenesis and tumor progression similarly to those described in other cancers. In conclusion, careful inclusion of diverse populations in (epi)genetic studies is integral for further understanding GEP-NEN biology, generalizing findings to diverse patient populations, and improving therapy for all.

## Supplementary Material

Figure S1 Assays used to analyze biomolecules in the 16 studies of GEP-NENs that reported patient race.

Figure S2 Comparison of racial group representation in GEP-NEN genomics, cancer genomics, and in the US population per 2020 census data.

Figure S3 Composition of Black and White patient cohorts from the AACR GENIE dataset by sequencing assay ID’s, depicted by raw subject numbers (Left) and by % composition (Right). Only sequencing assays that included both Black and White patients were considered.

Supplementary Data S1-S3 AACR Genie Comparisons (SD.1), STRING Interactions (SD.2), GO Enrichment of STRING data (SD.3)

Appendix 1 Structured Queries to PUBMED and EMBASE for Systematic Literature Review of GEP-NEN Genomics Studies.
