# Supplementary figures and images for: Under-Representation of Racial Groups in Genomics Studies of Gastroenteropancreatic Neuroendocrine Neoplasms
Source: Cancer Res Commun. 2022 Oct 12;2(10):1162–73. doi: 10.1158/2767-9764.CRC-22-0093 (PMC10035394; doi:10.1158/2767-9764.CRC-22-0093)

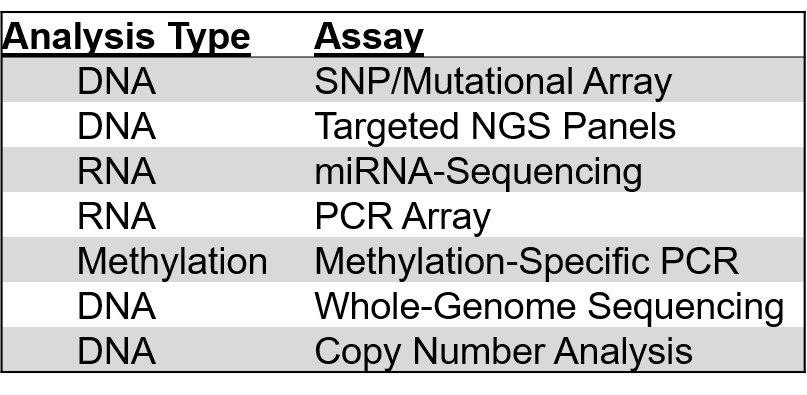


**Fig. S1) Assays Used in 16 Manuscripts Reporting Patient Race**

Supplement: Figure S1 — Assays used to analyze biomolecules in the 16 studies of GEP-NENs that reported patient race. [file crc-22-0093-s01.docx]

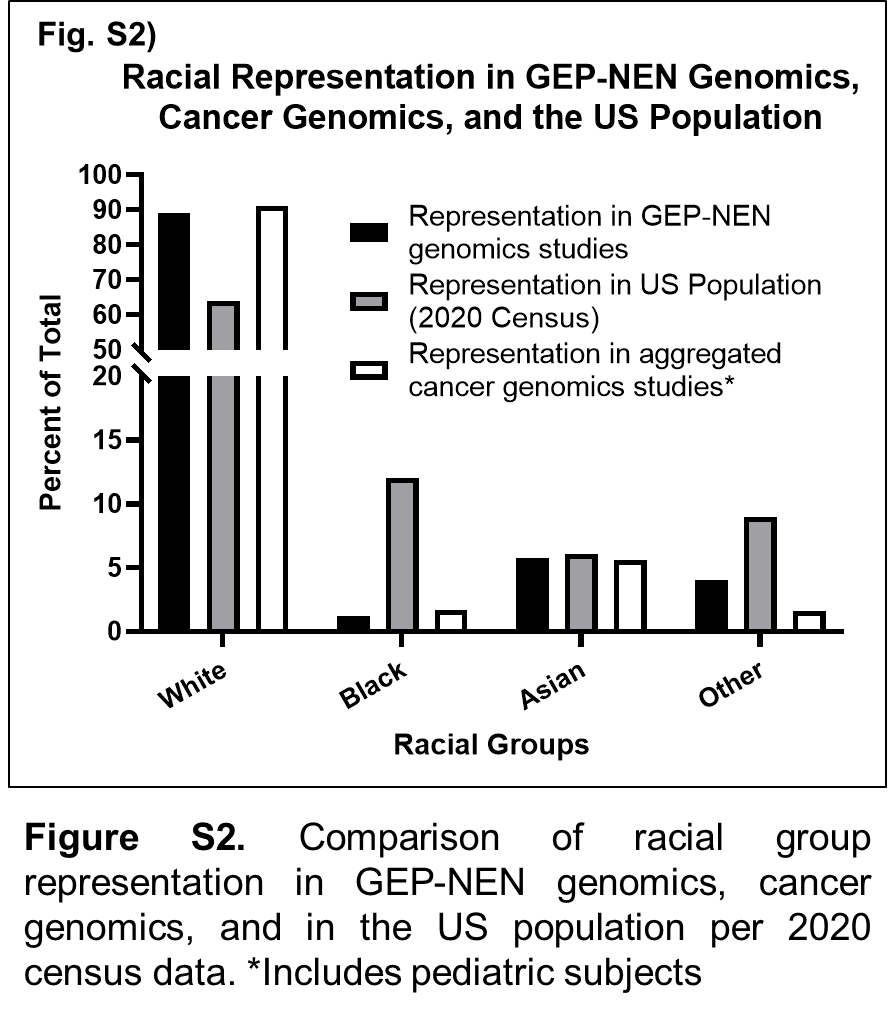

Supplement: Figure S2 — Comparison of racial group representation in GEP-NEN genomics, cancer genomics, and in the US population per 2020 census data. [file crc-22-0093-s02.docx]

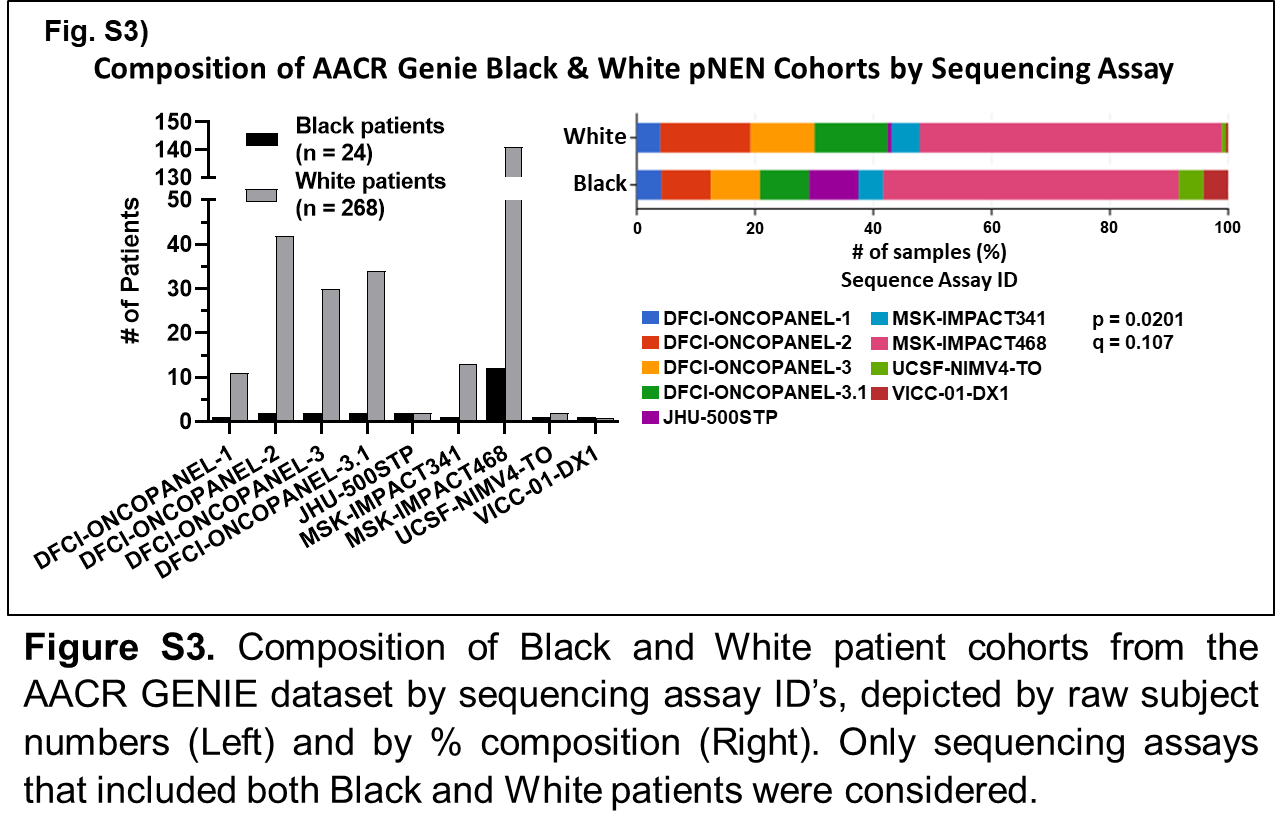

Supplement: Figure S3 — Composition of Black and White patient cohorts from the AACR GENIE dataset by sequencing assay ID’s, depicted by raw subject numbers (Left) and by % composition (Right). Only sequencing assays that included both Black and White patients were considered. [file crc-22-0093-s03.docx]
