# Supplementary material for: Under-Representation of Racial Groups in Genomics Studies of Gastroenteropancreatic Neuroendocrine Neoplasms
Source: Cancer Res Commun. 2022 Oct 12;2(10):1162–73. doi: 10.1158/2767-9764.CRC-22-0093 (PMC10035394; doi:10.1158/2767-9764.CRC-22-0093)
Supplement: Appendix 1 — Structured Queries to PUBMED and EMBASE for Systematic Literature Review of GEP-NEN Genomics Studies. [file crc-22-0093-s05.docx]

**Appendix 1. Literature Search Queries**

1. **PUBMED**

((genomics) OR (sequencing) OR (genetics) OR (epigenetics) OR (epigenomics) OR (transcriptomics)) AND ((gastroenteropancreatic neuroendocrine tumor) OR (pancreatic neuroendocrine tumor) OR (small bowel neuroendocrine tumor) OR (colon neuroendocrine tumor) OR (gastroenteropancreatic neuroendocrine cancer) OR (pancreatic neuroendocrine cancer) OR (small bowel neuroendocrine cancer) OR (colon neuroendocrine cancer) OR (gastroenteropancreatic neuroendocrine carcinoma) OR (pancreatic neuroendocrine carcinoma) OR (small bowel neuroendocrine carcinoma) OR (colon neuroendocrine carcinoma) OR (gastrointestinal carcinoid) OR (small bowel carcinoid))

1. **EMBASE**

(genomics OR sequencing OR genetics OR epigenetics OR epigenomics OR transcriptomics) AND (gastroenteropancreatic AND neuroendocrine AND tumor OR (pancreatic AND neuroendocrine AND tumor) OR (small AND bowel AND neuroendocrine AND tumor) OR (colon AND neuroendocrine AND tumor) OR (gastroenteropancreatic AND neuroendocrine AND cancer) OR (pancreatic AND neuroendocrine AND cancer) OR (small AND bowel AND neuroendocrine AND cancer) OR (colon AND neuroendocrine AND cancer) OR (gastroenteropancreatic AND neuroendocrine AND carcinoma) OR (pancreatic AND neuroendocrine AND carcinoma) OR (small AND bowel AND neuroendocrine AND carcinoma) OR (colon AND neuroendocrine AND carcinoma) OR (gastrointestinal AND carcinoid) OR (small AND bowel AND carcinoid)) AND ([article]/lim OR [article in press]/lim OR [conference paper]/lim OR [data papers]/lim OR [letter]/lim OR [note]/lim) AND [english]/lim AND [adult]/lim AND [embase]/lim AND [2000-2021]/py
